# Supplementary material for: A New Method for Determination of Thymol and Carvacrol in Thymi herba by Ultraperformance Convergence Chromatography (UPC2)
Source: Molecules. 2020 Jan 23;25(3):502. doi: 10.3390/molecules25030502 (PMC7037148; doi:10.3390/molecules25030502)
Supplement: Supplementary file 1 [file molecules-25-00502-s001.pdf]

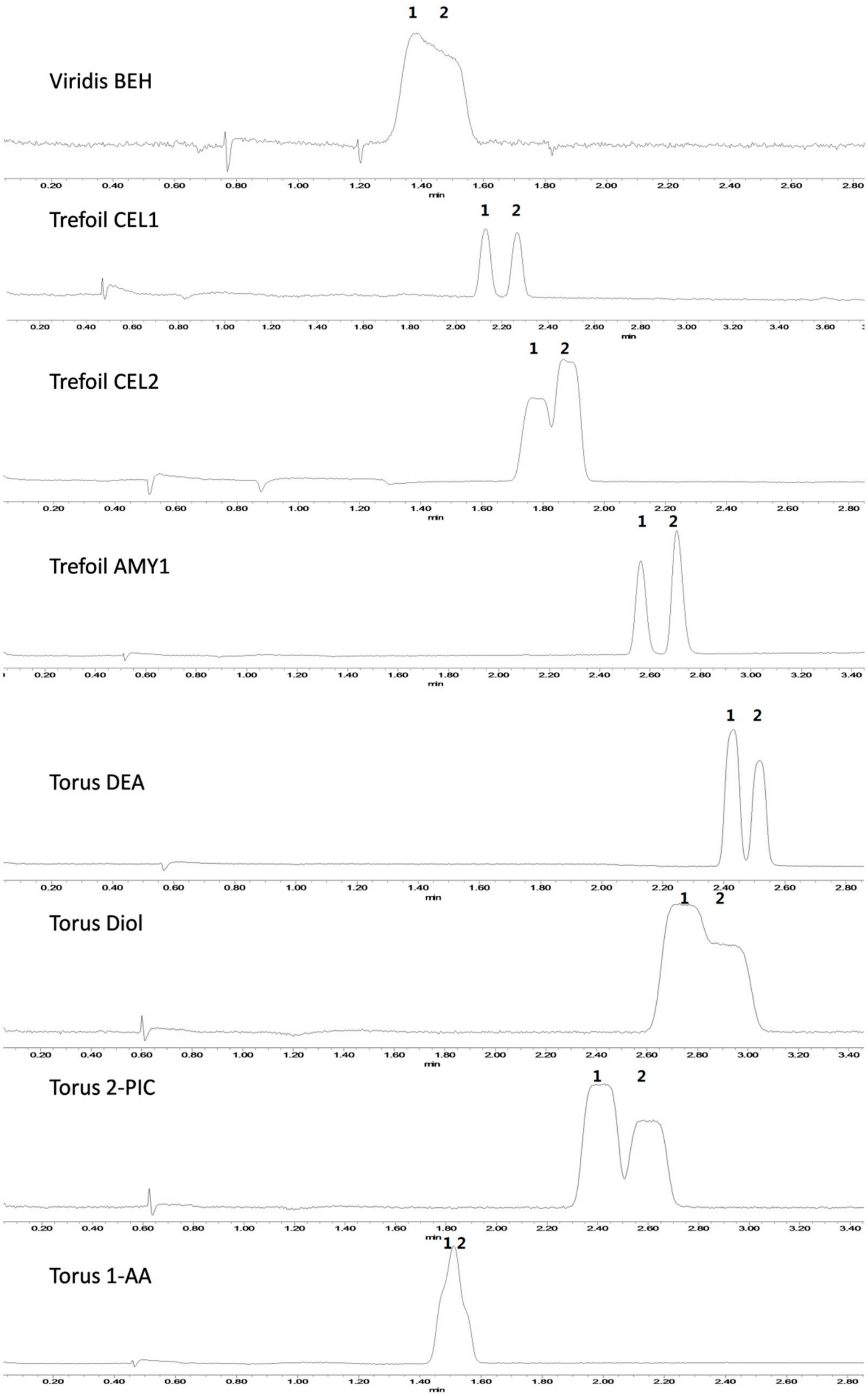

Figure 1. UPC^2 chromatograms for the separation of 2 isomers by different columns. 1 and 2 represent thymol (Thl) and carvacrol (Cal), respectively.Chromatographic conditions: A = CO2, B = Methanol. 1% B (initial), 1–3% B (0–1.5 min), 3–3% B (1.5–2.5 min), 3–15% B (2.5–10 min), column temperature = 30 °C, automated back pressure regulator (ABPR) = 1700 psi, flow rate = 1.0 mL/min, injection volume = 1.0 μL.

Discussion:

Since the purchase time of different chromatographic columns is different, the standard solutions used for each determination are prepared fresh, so the concentration may be different. It is gratifying that this does not affect the separation effect of chromatographic column.

Different columns purchased from Waters were investigated: Waters ACQUITY® UPC2™ Viridis BEH (3.0 mm × 100 mm, 1.7 μm), Trefoil CEL1 (2.1 mm × 150 mm, 2.5 μm), Trefoil CEL2 (2.1 mm × 150 mm, 2.5 μm), Trefoil AMY1 (2.1 mm × 150 mm, 2.5 μm), Torus DEA (2.1 mm × 100 mm, 1.7 μm), Torus Diol (2.1 mm × 100 mm, 1.7 μm), Torus 2-PIC (2.1 mm × 100 mm, 1.7 μm), and Torus 1-AA (2.1 mm × 100 mm, 1.7 μm) column. As can be observed, the Viridis BEH and Torus 1-AA columns cannot separate Thl and Cal at all; the Trefoil CEL2, Torus DEA, Torus Diol, and Torus 2-PIC columns detected Thl and Cal, but Thl and Cal peaks were not well separated; only the Trefoil CEL1 and Trefoil AMY1 columns detected Thl and Cal peaks were well separated. Notably, the Trefoil CEL1 column led to shorter analysis time, better resolution and peak shape (Figure S1). Therefore, Trefoil CEL1 column is selected as optimum column for further investigation.
